# Editorial: Roles and mechanisms of adipokines in metabolic diseases

**DOI:** 10.3389/fendo.2023.1303966

**Published:** 2023-11-06

**Authors:** Liwei Xie, Xi Li

**Affiliations:** ^1^ School of Public Health, Xinxiang Medical University, Xinxiang, China; ^2^ State Key Laboratory of Applied Microbiology Southern China, Guangdong Provincial Key Laboratory of Microbial Culture Collection and Application, Guangdong Open Laboratory of Applied Microbiology, Institute of Microbiology, Guangdong Academy of Sciences, Guangzhou, China; ^3^ The Institute of Life Science, School of Basic Medicine, Chongqing Medical University, Chongqing, China

**Keywords:** adipokine, adiponectin, metabolic diseases, vaspin, GDF15

Adipose tissue is not merely a storage depot for fat but serves as an active endocrine organ (1). It secretes a variety of bioactive molecules, including adipokines, cytokines, and other signaling factors that play a pivotal role in regulating systemic metabolism. Dysregulation of these adipokines is implicated in a host of metabolic disorders, including obesity, insulin resistance, type 2 diabetes, cardiovascular diseases, and other metabolic syndromes (2). This Research Topic features four seminal papers that shed new light on the intricate roles of adipokines, adipose tissue, and immune cells in metabolic health and type 2 diabetes.

Wang et al. offer a comprehensive review that delves into the complex crosstalk between adipose tissue and various other organs. They discuss the heterogeneity of adipose depots, which include subcutaneous, visceral, and brown fat. These depots contain adipocytes that not only interact locally with vasculature, nerves, and immune cells but also secrete hormones like leptin that have distal effects on organs such as the brain. This intricate inter-organ communication mediated by adipokines has far-reaching implications, affecting diverse physiological processes ranging from thermogenesis to inflammation. For example, adipokines have been shown to modulate insulin sensitivity in skeletal muscle and contribute to hepatic steatosis. An in-depth understanding of this crosstalk could unveil novel therapeutic targets for obesity, diabetes, and associated metabolic disorders.

Building upon this theme, Rapöhn et al. introduce a new human vaspin transgenic mouse model. Vaspin is an adipokine known for its insulin-sensitizing and anti-inflammatory properties. Unlike previous vaspin mouse models, this newly developed line exhibited circulating vaspin levels that were orders of magnitude higher than those in control mice. Intriguingly, despite these supraphysiological levels, the mice were protected against high-fat diet-induced obesity, hyperglycemia, and hypercholesterolemia. They also displayed elevated energy expenditure, which could potentially explain their resistance to weight gain. This innovative model serves as a valuable tool for further exploring the protective metabolic mechanisms mediated by vaspin *in vivo*.

In a clinical context, Zheng et al. investigated the potential of adipokine levels as biomarkers for metabolic disease risk stratification in middle-aged Chinese adults. They specifically evaluated the ratio of growth differentiation factor-15 (GDF15) to adiponectin and found it to be associated with metabolic syndrome in both genders. Interestingly, adiponectin alone was a better predictor of risk in men. These findings underscore the potential utility of circulating adipokines as early indicators of metabolic dysfunction.

Lastly, Li et al. employed Mendelian randomization to assess the causal role of immune cells in type 2 diabetes. Their findings indicate that higher genetically predicted monocyte counts and specific T lymphocyte subsets are associated with increased susceptibility to type 2 diabetes, thereby supporting the notion that immune components are integral to the pathogenesis of metabolic diseases.

In summary, these papers collectively advance our understanding of the multifaceted interplay between adipokines, metabolic health, and dysfunction. Elucidating the signaling mechanisms of adipokines could pave the way for the development of new biomarkers or targeted therapies for prevalent conditions like obesity, diabetes, and atherosclerosis. Future research avenues should focus on the discovering of new adipokines from beige and brow adipose tissue, the regulation of fat browning/beiging by adipokines, their effects on the gut microbiome, roles in hepatic steatosis, and impact on cardiovascular outcomes. Translating these findings from animal models to human clinical settings remains a critical next step. Large-scale longitudinal studies involving diverse populations will be essential to validate the clinical utility of adipokines as biomarkers. Ultimately, modulating adipokine signaling pathways may offer a promising therapeutic avenue for tackling obesity, insulin resistance, and type 2 diabetes.

## Author contributions

LX: Writing – original draft, Writing – review & editing. XL: Writing – original draft, Writing – review & editing.
